# CD5 Expression in CTCL and Its Implications for Anti-CD5 CAR T-Cell Therapy

**DOI:** 10.3390/ijms262110411

**Published:** 2025-10-27

**Authors:** Leena Wardeh, Madeline Williams, Courtney Prestwood, Zachary Wolner, Neda Nikbakht

**Affiliations:** 1 Department of Dermatology and Cutaneous Biology, Thomas Jefferson University, Philadelphia, PA 19107, USA; leena.wardeh@students.jefferson.edu (L.W.); madeline.williams@students.jefferson.edu (M.W.); 2 Department of Dermatology, Emory University, Atlanta, GA 30322, USA; courtney.prestwood@emory.edu (C.P.); zachary.wolner@emory.edu (Z.W.); 3 Winship Cancer Institute, Emory University, Atlanta, GA 30322, USA; 4 Department of Hematology and Medical Oncology, Emory University, Atlanta, GA 30322, USA

**Keywords:** CD5, cutaneous T-cell lymphoma, mycosis fungoides, single-cell RNA sequencing, tumor microenvironment, CAR T-cell therapy, antigen loss, T-cell immunotherapy, spatial transcriptomics

## Abstract

Cutaneous T-Cell Lymphomas (CTCL) are a heterogenous group of T-cell malignancies in the skin and have poor treatment outcomes in advanced stages. CD5, a surface glycoprotein expressed on most mature T cells, has emerged as a promising target for chimeric antigen receptor (CAR) T-cell therapy in systemic T-cell lymphomas. However, its expression profile in CTCL and relevance for targeted therapy remain unclear. Notably, in CTCL, the cell surface expression of receptors, such as CD7 and CD26, tends to become downregulated on the surfaces of malignant T cells In this study, we analyzed single-cell RNA sequencing (scRNA-seq) data from patients at two institutions with mycosis fungoides (MF), the most common subtype of CTCL with a predominantly CD4 phenotype. We utilized 5 patch/plaque MF skin biopsies (majority from early-stage patients), 8 MF tumor biopsies (all from advanced-stage patients), and 8 healthy control biopsies to evaluate lesion-specific *CD5* gene expression on CD4 T cells. We found that *CD5* was significantly increased in malignant MF CD4 T cells compared to healthy control CD4 T cells (21.1% of MF CD4 T cells expressed *CD5* vs. 5.2% of healthy control CD4 T cells, respectively). In subgroup analysis, patch/plaque stage MF biopsies showed higher expression of *CD5* in CD4 T cells than tumor stage MF biopsies. Notably, 94.3% of malignant CD4^+^ T cells in tumor stage MF lesions exhibited complete *CD5* loss compared to only 76.6% in patch-plaque MF lesions, suggesting antigen escape in tumor stage disease. These findings demonstrate that *CD5* expression in CTCL is dynamic and varies based on lesion type. Our work suggests CD5 may be a viable therapeutic target in MF with patch/plaque presentations but may not be as effective in advanced stages of MF with tumor presentations. This work informs *CD5* gene expression in MF based on clinical lesion type and further information is needed to clarify clinical implications as a future therapeutic target.

## 1. Introduction

Cutaneous T-cell lymphomas (CTCL) are a group of rare non-Hodgkin lymphomas characterized by the proliferation of malignant T-cells in the skin. The most common subtype, mycosis fungoides (MF), primarily involves the skin but may progress to lymph nodes, peripheral blood, and visceral organs [1,2]. Although early-stage disease often presents with an indolent course, advanced-stage CTCL is associated with limited treatment options and poor overall survival [3].

CD5 is a transmembrane glycoprotein expressed on thymocytes, mature T-cells, and a subset of B-cells. It plays a critical role in regulating T-cell receptor signaling, modulating immune activation, and maintaining peripheral tolerance [4,5]. In systemic mature T-cell lymphomas, CD5 expression has been associated with higher clinical stage, bone marrow involvement, and poorer outcomes [6]. However, CTCL was excluded from these analyses, and the clinical relevance of CD5 expression in CTCL remains poorly described. Notably, CTCL frequently exhibits downregulation or complete loss of surface antigens including CD26 and CD7, particularly in advanced disease stages [7,8]. Cell surface antigen modification is a recognized mechanism of immune evasion and therapeutic resistance, and it raises concerns that CD5 downregulation may impact the success of CD5-targeted therapies in CTCL.

The development of CD5-directed chimeric antigen receptor (CAR) T-cell therapy for relapsed and refractory T-cell lymphomas has generated substantial interest. Early-phase studies have shown clinical response rates of approximately 60% in systemic T-cell lymphomas, with manageable toxicity profiles [8,9,10,11,12]. However, the lack of data on CD5 expression patterns in CTCL, combined with the known prevalence of antigen loss in this disease, presents an opportunity to evaluate CD5 expression and its relationship to the outcome of CD5-directed CAR T-cell therapy in this patient population.

To better understand the role of CD5 in CTCL and evaluate its potential as a therapeutic agent, we assessed *CD5* gene expression on malignant CD4 T cells in MF patients utilizing single-cell RNA sequencing (scRNA-seq) strategies. Our study aims to inform the use of CD5-targeted immunotherapies in CTCL and guide patient selection for future clinical trials.

## 2. Results

### 2.1. Written Results

#### 2.1.1. CD4 T Cells Are Prominent in MF Skin Lesions

**Figure 1:**
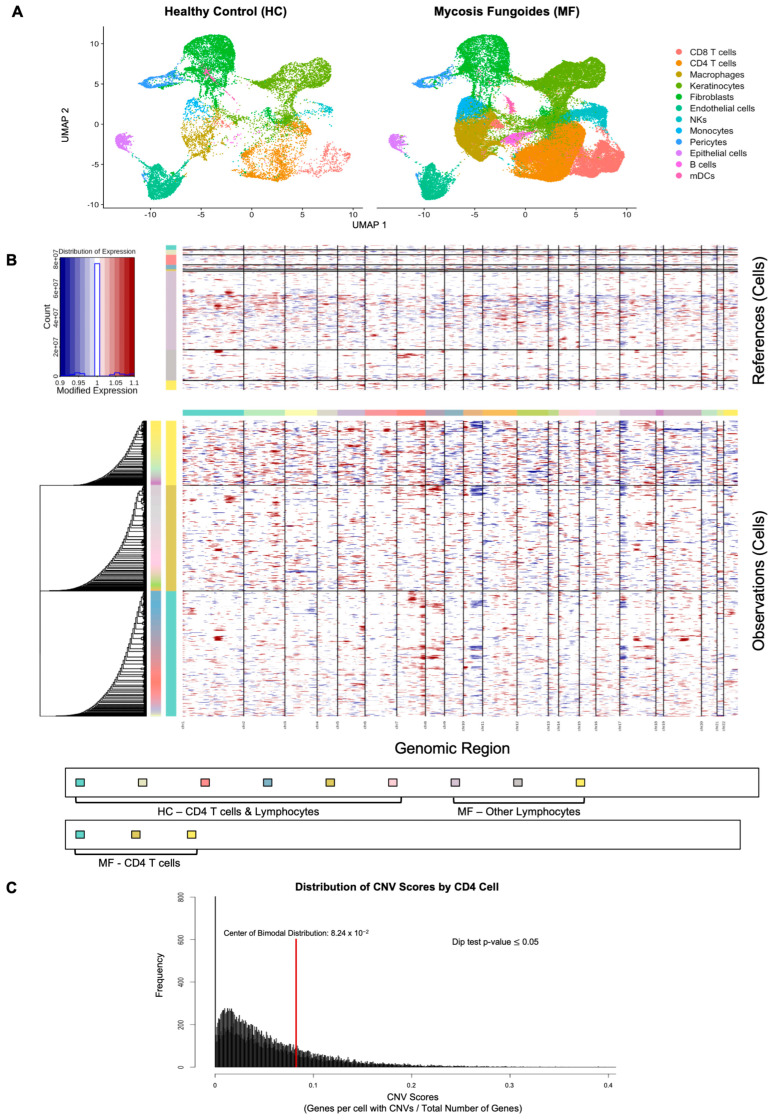
Cell Clustering, and Genomic Instability/CNV burden in MF and Healthy Control Lymphocytes. (**A**) UMAP projection of all integrated samples, showing clustering of major cell types in healthy control skin (**left**) and Mycosis Fungoides (MF) lesions (**right**). Cell types are annotated by canonical markers, including CD4 T cells, CD8 T cells, keratinocytes, fibroblasts, and myeloid populations. (**B**) Heat map of InferCNV results. Top panel indicates reference lymphocytes from healthy controls and MF patients, and bottom panel shows CD4 T cells from MF patients. Genomic amplifications by chromosome (*x*-axis) are shown in red and genomic deletions are shown in blue. (**C**) Density plot showing number of CD4 T cells by CNV burden (i.e., number of genes with CNVs per cell). Red line indicates center of bimodal distribution and cutoff for determining malignant vs. healthy CD4 T cells.

We compared scRNA-seq data obtained from the skin of MF patients with either early-stage patch/plaque or advanced-stage tumor lesions to healthy control skin. To identify the prominent cell populations in the MF skin lesions, we analyzed publicly available scRNA-seq data from the University of Vienna and University of Pittsburgh [13,14,15,16,17]. We compared scRNA-seq data collected from skin of healthy individuals (*n* = 4) to that of CTCL skin lesions (*n* = 13; patch/plaque lesions = 5, tumors = 8) (Table 1). 81,124 cells from these 17 samples passed quality control and were resolved into various cell clusters based on canonical cell markers and differential expression testing (Table 2). To identify CD4 T-cells, including the presumed malignant CD4 T cells in the MF samples, the differential expression of *CD3D*, *IL7R*, and *CCR7* among other canonical CD4 T cell gene signatures was used (Table 2). The uniform manifold approximation and projection (UMAP) resolved 12 cell clusters in healthy control and MF skin (Figure 1A). As expected, compared to healthy control skin, the CD4 T cell population was the prominent cell population in MF skin lesions (Figure 1A).

#### 2.1.2. Malignant CD4 Cell Identification by Copy Number Variants

**Figure 2:**
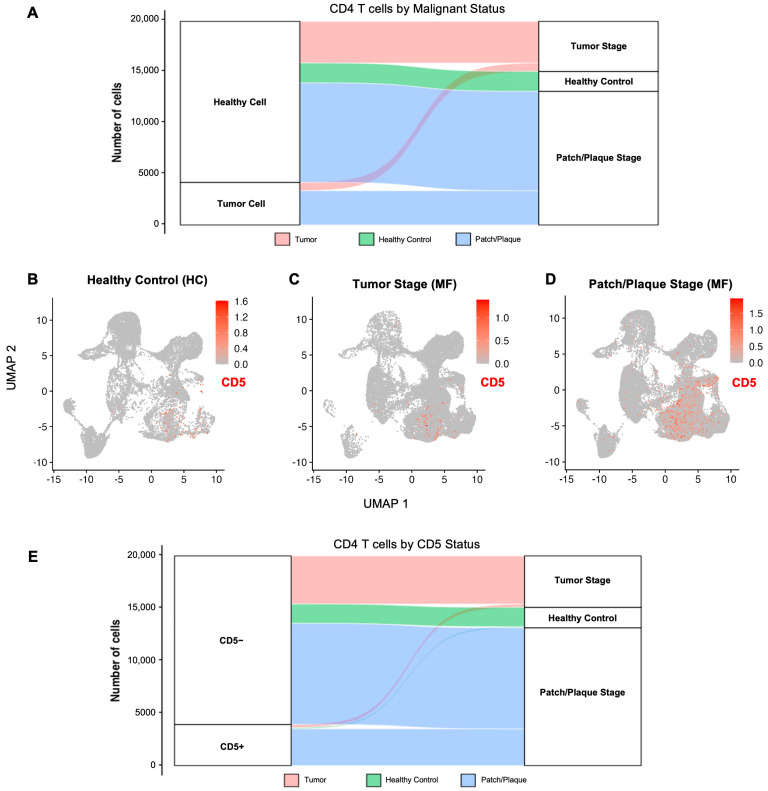
CD5 expression across disease stages in cutaneous T-cell lymphoma (CTCL) and healthy skin by single-cell RNA sequencing. (**A**) Alluvial plot showing malignant status determined by CNV burden across CD4 T cells from tumor-stage MF lesions (red), patch/plaque MF lesions (blue), and healthy controls (green). (**B**–**D**) CD5 expression feature plots highlighting CD5-expressing cells (expression intensity scale: red = more CD5 expression, grey = no CD5 expression) in healthy controls (**B**), tumor-stage MF lesions (**C**), and patch/plaque-stage MF lesions (**D**). (**E**) Alluvial plot showing number of CD4 T cells by CD5+ versus CD5− status from tumor-stage MF lesions (red), patch/plaque MF lesions (blue), and healthy controls (green).

We identified malignant cells within MF samples using chromosomal copy number variant (CNV) burden. These CNV profiles for MF cells and HC cells (used as reference cells in analysis) were visualized in a heatmap (Figure 1B). After scoring each cell for their CNV burden (i.e., number of genes with CNVs per cell), we found a significant bimodal distribution (Figure 1C). Malignant cells were then defined as those with CNV signal above 0.082. Using this method, a total of 4168 malignant CD4 cells were identified in MF samples (Figure 2A).

#### 2.1.3. *CD5* Expression Across All Cell Clusters

Next, we assessed *CD5* expression in all cell clusters from healthy control, tumor-stage, and patch/plaque stage MF lesions. Amongst all cell clusters identified across all groups, CD4 T cells had the highest *CD5* expression, with CD8 T cells and natural killer cells showing less, but still observable expression of *CD5* (Figure 2B–D). Minimal *CD5* expression was observed in keratinocytes, fibroblasts, myeloid cells, or other non-T-cell compartments, supporting the specificity of *CD5* expression to the lymphocytic cells in CTCL, especially the CD4 T cells.

#### 2.1.4. *CD5* Expression in CD4 T Cells from MF Patients Versus Healthy Controls

Of the 19,909 CD4 T cells identified across all groups and the 4168 that were malignant, *CD5* expression was significantly increased in malignant CD4 T cells from all MF patients (log_2_ fold change of 1.85, adjusted *p*-value < 0.0001), indicating elevated *CD5* expression in malignant MF CD4 T-cells relative to healthy skin (Figure 2E). However, when MF cells were divided by lesion type and compared to healthy control CD4 T cells, only malignant CD4 T cells from patch/plaque lesions showed significantly different *CD5* expression compared to healthy CD4 T cells (log_2_ fold change of 2.10, adjusted *p*-value < 0.0001). Malignant CD4 cells from tumor-stage lesions did not have significantly different CD5 expression compared to healthy controls (log_2_ fold change of −0.24, adjusted *p*-value > 0.05).

#### 2.1.5. *CD5* Expression in Malignant CD4 T Cells from Patch/Plaque Versus Tumor-Stage MF Lesions

*CD5* expression was significantly reduced in malignant CD4 T cells from tumor-stage MF lesions compared to patch/plaque MF lesions (Figure 2B–E). Malignant CD4 T-cells from tumor lesions exhibited a log_2_ fold change of −2.34 (adjusted *p*-value < 0.0001). Notably, approximately 94.3% of malignant CD4 T-cells in tumor-stage lesions did not express *CD5*, compared to only 76.6% of malignant CD4 T cells from patch/plaque MF lesions. This suggests the emergence of CD5-negative malignant subpopulations in MF tumor lesions.

#### 2.1.6. CD5 Expression in Other Lymphocytes in MF

Of the 8289 CD8 T cells identified, *CD5* expression was not significantly different in CD8 T cells from MF patients compared to healthy controls (adjusted *p*-value > 0.05). There were also 3224 natural killer cells identified, and *CD5* expression was not significantly different between natural killer cells from MF patients compared to healthy controls (adjusted *p*-value > 0.05).

## 3. Discussion

This study demonstrates that CD5 expression in CTCL is largely dependent on lesion type, with malignant CD4 T cells from patch/plaque MF lesions showing elevated *CD5* transcript levels compared to malignant CD4 T cells from MF tumor lesions and healthy control CD4 T cells. These findings provide a high-resolution view of CD5 heterogeneity and support the idea that its therapeutic potential may be influenced by MF lesion type.

The observed decrease in *CD5* expression in tumor-stage MF lesions aligns with earlier findings that progressive surface antigen loss is a mechanism of immune evasion and treatment resistance in CTCL [7]. Similar patterns have been reported with CD7 and CD26 in CTCL, where antigen loss limits the durability of targeted therapies [8]. Our findings extend this paradigm to CD5 and support the notion that CD5 downregulation may be another form of immune evasion in CTCL.

CD5-directed CAR T-cell therapy has shown promising antitumor activity in systemic T-cell lymphomas, with overall response rates approaching 60 percent and manageable toxicity [8,9,10,11,12]. Recent studies in systemic mature T-cell lymphomas, such as those by Elghawy et al. [6], demonstrated that CD5 expression is prevalent in approximately 63 percent of cases and is associated with worse survival outcomes. However, their analysis excluded CTCL, leaving uncertainty around the role of CD5 in this distinct subtype. Our data begins to fill this gap, showing that CD5 is indeed highly expressed in malignant CTCL CD4 T cells, particularly in patch/plaque lesions, but that expression is heterogeneous and can be lost in more robust MF tumors.

In the cells analyzed, we observed that *CD5* expression was primarily restricted to lymphocytes, including malignant CD4 T cells which had the highest expression, and was not detected in other populations such as keratinocytes, fibroblasts, or myeloid cells. It is worth noting that this study is limited to surveying *CD5* expression in the skin, not the blood of CTCL patients. It remains to be seen if similar downregulation of CD5 occurs in the blood compartment of patients with leukemic MF and advanced CTCL.

The limitations of this study include its use of public data, the small sample size, and the inability to assess other tissues (blood and lymph nodes). The use of a small sample size in one tissue compartment limits the broader applicability of our conclusions but indicates a need for future research addressing CD5 expression beyond the skin in MF patients. Additionally, while we used CNV burden to distinguish malignant CD4 T cells in MF samples, T-cell clonality was not utilized to confirm the identity of these malignant clones. Future directions include validating these transcriptomic findings at the protein level through multiplex IHC and spatial transcriptomics, as well as conducting longitudinal studies to track CD5 expression changes over the course of disease progression.

In summary, this study identifies a dynamic, lesion-specific pattern of *CD5* expression in CTCL and supports that CD5 may be downregulated in skin lesions of advanced stage MF patients. These findings underscore the importance of biomarker-driven patient selection for CD5 CAR T-cell therapy and the need for therapeutic strategies that account for antigen heterogeneity.

## 4. Materials and Methods

### 4.1. Sample Selection and Data Acquisition

Single-cell RNA sequencing (scRNA-seq) data from patients with Mycosis Fungoides (MF) and healthy control skin were obtained from publicly available Gene Expression Omnibus (GEO) repositories. Datasets from the University of Vienna (cases 1–12) and University of Pittsburgh (cases 13–17) included 5 classic patch/plaque-stage MF samples, 8 tumor-stage MF samples, and 8 healthy control samples (Table 1) [13,14,15,16,17]. GEO accession numbers included GSE128531, GSM5280111, GSM5047045, GSM5261812, GSM5534588, GSM5534589, and GSM8255139–GSM8255145. Sample metadata and clinical annotations were reviewed to ensure inclusion of representative patch/plaque and tumor lesions.

### 4.2. Computational Environment

All analyses were conducted using R (version 4.4.1, R Foundation for Statistical Computing, Vienna, Austria) and RStudio (version 2024.9.0.375, Posit Software, PBC, Boston, MA, USA). The Seurat package version 5.1 was used for preprocessing, normalization, integration, clustering, and visualization. Additional packages included SCTransform (v0.4.1) for normalization and variance stabilization [18,19,20].

### 4.3. Preprocessing and Quality Control

Cells were retained if they expressed between 200 and 6000 genes and had fewer than 5% mitochondrial transcripts, minimizing inclusion of dead or low-quality cells. In total, 81,124 high-quality single cells were retained for analysis (Table 2).

### 4.4. Normalization and Feature Selection

Data were normalized using SCTransform (v2), with the number of variable features set to 2000. This approach stabilizes technical variance while preserving the biological signal.

### 4.5. Data Integration and Clustering

Samples were integrated using Seurat’s canonical correlation analysis (CCA)–based anchor integration to align datasets from different conditions. Dimensionality reduction was performed using principal component analysis (PCA) followed by Uniform Manifold Approximation and Projection (UMAP) for visualization. Using the ElbowPlot function in Seurat, a total of 12 distinct clusters were identified.

### 4.6. Cell Type Annotation

Clusters were annotated using canonical gene markers (Table 2). CD4 T cells were identified using *CD3D*, *IL7R*, and *CCR7* expression. Other cell types such as keratinocytes (*KRT1*, *KRT5*, *KRT14*), fibroblasts (*COL1A1*), myeloid cells (*LYZ*, *CD14*), and B cells (*MS4A1*) were annotated based on established lineage markers. Cell population counts by cluster are included in Table 2.

### 4.7. Estimating and Inferring Malignant CD4 T Cells

The CNV level of each cell was obtained using InferCNV in R (v1.20.0). Healthy control lymphocytes were used as reference cells (B cells, NKs, CD8 T cells, and CD4 T cells) in addition to B cells, NKs, and CD8 T cells from MF patients. The R code is available at https://github.com/broadinstitute/inferCNV (accessed 13 October 2025) and was used with default settings, except for a cutoff of 0.1. Cells were sorted based on the number of genes per cell with CNVs and stratified based on the resulting bimodal distribution. Malignant cells were defined as having CNVs in greater than 8.24% of genes.

### 4.8. Differential Gene Expression Analysis

Differential expression of *CD5* (ENSG00000110448) was evaluated using the Wilcoxon Rank-Sum test with Bonferroni correction for multiple hypothesis testing. An expression cutoff >0 UMI was used to classify cells as CD5 positive. Comparisons were performed between MF and healthy control CD4 T cells, and between patch/plaque-stage and tumor-stage MF lesions. Genes with adjusted *p*-values < 0.05 were considered statistically significant.

### 4.9. Visualization

UMAP projections and alluvial plots were used to visualize *CD5* expression across disease states. All plots were generated using Seurat.

## Figures and Tables

**Table 1:** CTCL and healthy control patient cases included in scRNA-seq analysis. Case number, GEO IDs, lesion type, clinical and pathological stages, and large cell transformation (LCT) status for each patient biopsy included in the study.

Cases of CTCL Used for scRNA-Seq				
Case No.	GEO ID	Lesion	Clinical Stage	Pathological Stage
1	GSM5280111	Patch/Plaque	IB	T2bN0M0B0
2	GSM5047045	Patch/Plaque	IVB	T3N3M1B1
3	GSM5261812	Patch/Plaque	IIB	T2bN0M0B0
4	GSM5534588	Patch/Plaque	IA	T1bN0M0B0
5	GSM5534589	Patch/Plaque	IA	T1aN0M0B0
6	GSM8255139	Healthy Control	-	-
7	GSM8255140	Healthy Control	-	-
8	GSM8255141	Healthy Control	-	-
9	GSM8255142	Healthy Control	-	-
10	GSM8255143	Tumor	IVA	TNM Unknown, B2
11	GSM8255144	Tumor	IVA	TNM Unknown, B2
12	GSM8255145	Tumor	IIB	TNM Unknown, B0
13	GSM3679033	Tumor	IVA	T4NxB2M0
14	GSM3679034	Tumor	IIB	T3N0B0M0
15	GSM3679035	Tumor	IIB	T3NxB0M0
16	GSM3679036	Tumor	IIB	T3N0B0M0
17	GSM3679037	Tumor	IVA	T4NxM0B0

**Table 2:** Canonical gene markers used for cell type annotation. List of cell types profiled in the scRNA-seq dataset and the specific gene markers used to classify each population. Includes T-cell, myeloid, epithelial, and stromal cell types, as well as the total number of annotated cells per lineage.

Canonical Markers for scRNA-Seq		
Cell Type	Marker	No. of Cells
B cells	*MS4A1*	977
Endothelial cells	*PECAM1*	5447
Epithelial cels	*KRT18*	1596
Fibroblasts	*COL1A1*	10,930
Keratinocytes	*KRT1, KRT5, KRT14*	17,851
NKs	*GNLY*	3224
Macrophages	*CD16, LYZ*	7887
Monocytes	*CD14, LYZ*	2421
Pericytes	*RGS5*	1805
CD4 T cells	*CD3D, IL7R, CCR7*	19,909
CD8 T cells	*CD8A*	8289
mDCs	*CD83*	788

## Data Availability

The single-cell RNA sequencing datasets presented in the study are from prior studies and openly available on Gene Expression Omnibus (GEO) under GSE128531 and related GEO accessions.

## Abbreviations

The following abbreviations are used in this manuscript:

CTCL	Cutaneous T-cell lymphoma
MF	Mycosis Fungoides
CAR	Chimeric Antigen Receptor
scRNA-seq	Single-cell RNA sequencing
UMAP	Uniform Manifold Approximation and Projection
UMI	Unique Molecular Identifier
GEO	Gene Expression Omnibus
IHC	Immunohistochemistry
CD	Cluster of Differentiation
mDC	Myeloid Dendritic Cell
PCA	Principal Component Analysis
CCA	Canonical Correlation Analysis
HC	Healthy Control
RNA	Ribonucleic Acid
R	Statistical computing language R
